# Hypoglossal Nerve Palsy Following Cervical Spine Surgery—Two Case Reports and a Systematic Review of the Literature

**DOI:** 10.3390/brainsci15030256

**Published:** 2025-02-27

**Authors:** Felicia Hellquist, Victor Gabriel El-Hajj, Ali Buwaider, Erik Edström, Adrian Elmi-Terander

**Affiliations:** 1 Department of Medical Sciences, Örebro University, 701 82 Örebro, Sweden; 2 Department of Clinical Neuroscience, Karolinska Institutet, 171 77 Stockholm, Sweden; 3 Capio Spine Center Stockholm, Löwenströmska Hospital, 194 45 Upplands-Väsby, Sweden; 4 Department of Surgical Sciences, Uppsala University, 753 10 Uppsala, Sweden

**Keywords:** hypoglossal nerve palsy, HNP, cervical spine, spine surgery, anterior approach, posterior approach, complications

## Abstract

Background/Objectives: Hypoglossal nerve palsy (HNP) is a rare complication after cervical spine surgery and is reported after both anterior and posterior approaches. It often presents with dysarthria, dysphagia, and hoarseness. We present a systematic review of the literature and two cases of patients presenting with confirmed HNP after anterior cervical spine surgery. Methods: Two retrospective case reports and a systematic review of the literature were presented. The electronic databases PubMed and Web of Science were systematically searched from inception. Results: In total, 17 cases of HNP were reported in the literature, including the two hereby presented. Ten cases involved the anterior approach and seven the posterior approach. The reported risk of HNP following cervical spine surgery varied between 0.01% and 2.5% depending on the procedure. The main etiology was mechanical compression of the nerve. Most of the cases recovered within a few months with conservative treatment. In some cases, permanent hypoglossal injury with persistent symptoms was reported. In both of the current cases, the symptoms gradually improved and completely resolved after a few months. Conclusions: HNP is a rare complication after cervical spine surgery. The causes of hypoglossal palsy are multifactorial, but mechanical injury is the most common. A thorough understanding of the nerve’s anatomy is essential to minimize the risk of injury during anesthesia, patient positioning, and surgery. Understanding the underlying mechanisms contributing to HNP post-cervical spine surgery enables the implementation of preventive measures to mitigate its occurrence.

## 1. Introduction

Hypoglossal nerve palsy (HNP) is a rare condition, primarily related to tumors encroaching on the nerve [1]. In rare cases, HNP can manifest as a complication following cervical spine surgery [2]. Common symptoms of HNP include hoarseness, dysphagia, and dysarthria [2]. These symptoms not only cause a substantial level of discomfort to the patient but also correlate to other complications, such as aspiration pneumonia and weight loss [2]. The frequency of cervical spine procedures has grown steadily over the last decades, and a continued increase is predicted to align with the increased life expectancy [3]. It is therefore important to recognize the possible surgical risks and associated adverse events that may occur [4]. Understanding the underlying mechanisms contributing to HNP post-cervical spine surgery enables the implementation of preventive measures to mitigate its occurrence. This study aimed to illustrate two cases of HNP following cervical spine surgery and to review current literature on this condition. The primary variables of interest included mechanism of injury, possible treatment, follow-up, and outcomes.

## 2. Materials and Methods

### 2.1. Case Report

To compile the information for the case reports, data were collected and extracted from the electronic medical records. The records included details about clinical history, surgical reports, referrals to an ENT specialist, and visit notes. Informed consent was obtained from the patients.

### 2.2. Systematic Review of the Literature, Search Strategy, and Study Selection

The literature was then systematically searched for studies describing HNP following spine surgery. PubMed and Web of Science databases were searched to identify articles detailing the risk, etiology, and prognosis of HNP post-cervical spine surgery. With the assistance of a trained librarian, a search strategy for HN injury and surgical complications was devised (Table S1). Subsequently, the identified articles were organized using the Rayyan tool and screened for inclusion based on their titles and abstracts [5]. Duplicates were identified and eliminated in the software. A rigorous assessment based on titles and abstracts was performed in relation to the inclusion and exclusion criteria by two independent reviewers (F.H. and V.E.-H.). The remaining articles underwent full-text reviews by three blinded reviewers. Case reports and articles documenting hypoglossal palsy after cervical spine surgery were eligible for inclusion in the study, with no restrictions regarding the year of publication. Animal studies, thoracic or lumbar spine surgeries, hypoglossal palsy attributed to causes other than cervical spine surgery, letters, and systematic reviews were deliberately excluded from the systematic review. Articles not written in the English language were also excluded. The protocol applied is similar to that of previously published works (registration ID: CRD42022330809) [6,7,8,9]. This systematic review was conducted following the Preferred Reporting Items for Systematic Reviews and Meta-Analyses (PRISMA) guidelines.

### 2.3. Data Extraction

Variables of interest included study characteristics, number of HNP cases, surgical procedures, indications for surgical procedures, number of levels operated, severity of HNP, postoperative symptoms, follow-up time, and plausible causes. Recorded study data included author, study design, year of publication, and country. The severity of HNP was classified as permanent or temporary. The primary outcomes of interest were risk, plausible causes, and prognosis.

## 3. Results

### 3.1. This Case Report

#### 3.1.1. Case 1

**Figure 1:**
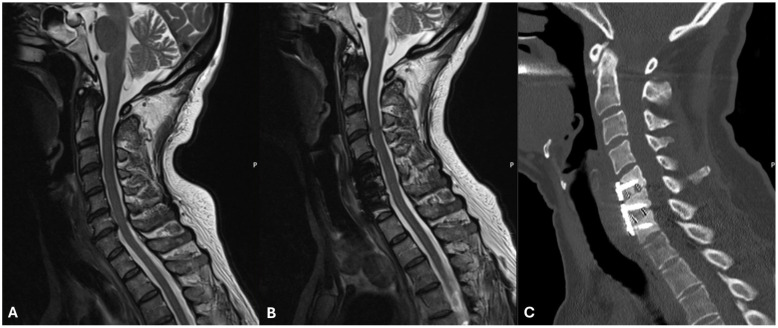
Preoperative MRI (**A**), as well as postoperative MRI (**B**) and CT (**C**) for case 1.

A previously healthy 55-year-old woman with no prior cervical surgery or any comorbidities presented with hoarseness and hypoglossal palsy after a right-sided two-level ACDF surgery [10,11,12]. Preoperatively, the patient presented with a 5-year history of intermittent neck pain and a radiating pain to the right arm and hand. Magnetic resonance imaging (MRI) showed intervertebral disc degeneration at C5–C6 and C6–C7 levels (Figure 1). Initial conservative treatment included physiotherapy and medication with Pregabalin. As a result of aggravated symptoms with constant radiculopathy, the patient underwent a two-level ACDF. Postoperatively, the patient presented with hoarseness, which was initially attributed to the use of a new reinforced endotracheal tube. Due to persistent complaints, the patient was referred to and examined by an ear, nose, and throat (ENT) specialist. Upon examination, a right-sided hypoglossal palsy with tongue deviation, along with a right-sided vocal cord paralysis, likely the result of a concomitant recurrent nerve palsy, was detected. Postoperative MRI did not show any relevant pathology. The symptoms gradually and spontaneously improved, with complete resolution observed at around three months postoperatively (Figure 1).

#### 3.1.2. Case 2

A previously healthy 55-year-old male, with no significant comorbidities and no history of prior spinal surgeries, presented with new-onset hoarseness, dysphagia, and right-sided hypoglossal palsy, following ACDF surgery. Preoperatively, the patient had experienced a 5-year history of intermittent neck pain, radicular symptoms radiating into the arms, and worsening difficulty. MRI imaging prior to surgery revealed significant cervical spine degeneration and central stenosis at the C5–C6 level with concomitant myelomalacia.

The patient underwent a one-level right-sided ACDF to address the degenerative changes and alleviate his symptoms. Postoperatively, the patient developed unexpected dysarthria, dysphagia, and hoarseness. Notably, he also exhibited signs of both recurrent nerve and HNP, which were confirmed through clinical examination and consultation with an ENT specialist. The HNP was characterized by right-sided weakness of the tongue, affecting his ability to speak and swallow effectively.

Postoperative MRI did not reveal any new or relevant pathology that could explain the HNP. The patient’s symptoms were managed with a combination of conservative approaches, including speech therapy and supportive care. Over time, there was gradual improvement in his condition. The dysphagia and hoarseness slowly resolved, and the hypoglossal nerve function gradually returned to normal within three months. The patient’s overall recovery was monitored closely to ensure complete resolution of the postoperative complications.

### 3.2. Review of Literature

**Figure 2:**
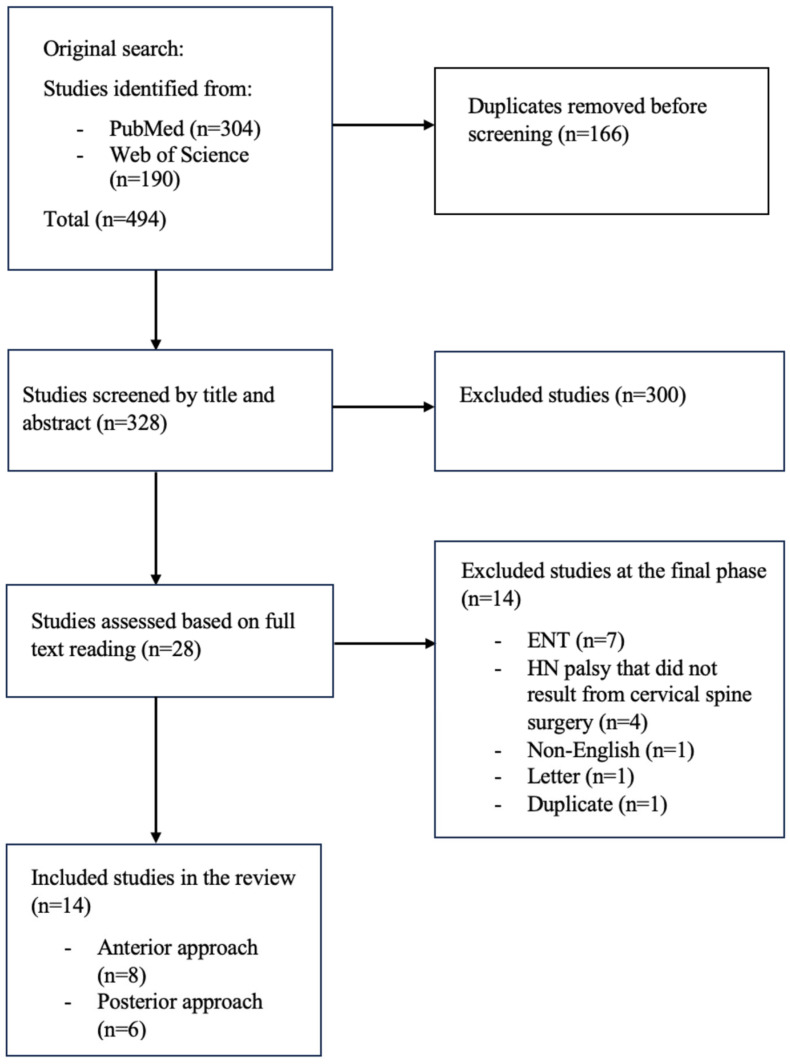
PRISMA Flowchart showing the number of studies included.

The search strategy provided 494 studies. After removal of duplicates, the remaining 328 were screened based on titles and abstracts. The remaining 28 underwent a full text review. Ultimately, 14 articles remained (Figure 2). Apart from the two current cases, a total of 15 cases of HNP were reported in the 14 studies, with eight reporting HNP following an anterior approach (Table 1) and seven reporting HNP following a posterior approach (Table 2). Age ranged from 35 to 67 years old. Based on the 12 patients for whom this information was available, the mean age was 46 years in the anterior group and 55 years in the posterior group. Ten out of 15 patients were male, while two were female [13,14]. In three of the studies [15,16,17], patient sex was not disclosed.

#### 3.2.1. HNP in Anterior Cervical Spine Surgery

The anterior surgeries included anterior cervical discectomy and fusion (*ACDF*), anterior cervical corpectomy and fusion (*ACCF*), and osteophyte resection with the Smith-Robinson approach [13,16,17,18,19,20,21,22]. One to three levels were operated on between the C2 and C7 vertebrae [13,16,17,18,19,20,21,22]. The indications for the surgeries varied from degenerative disorders (e.g., radiculopathy or myelopathy) [16,17,18,19] to trauma [20,22] and infection [13]. Yasuda et al. used the anterior approach for osteophyte removal in a patient with related dysphagia [21].

All reports agreed on the fact that HNP is a rare complication following anterior cervical spine surgery. A retrospective study by Yamagata et al. [16] and a prospective study by Saunders et al. [17] reported the risk after anterior surgery at 1/152 (0.66%) and 1/40 (2.5%), respectively.

The cause of injury most commonly suspected after the anterior approach was mechanical trauma to the hypoglossal nerve [13,18,19,20,21,22]. López et al. [20], Yasuda et al. [21], Sengupta et al. [13], and Park et al. [22] reported that excessive retraction of the hypoglossal nerve led to mechanical injury. Colón et al. [18] observed a surgical staple attached to the HN, which injured the nerve. Moreover, airway management by anesthesia was described as a possible reason for the nerve injury. Lee et al. [19] cited mechanical stress, hypotension, ischemia, neck positioning, reperfusion injuries, or surgical manipulation as potential causes. Yamagata et al. reported an anatomical anomaly of the hypoglossal nerve as a possible cause of nerve injury [16].

Symptoms that occurred immediately after surgery included dysarthria, dysphagia, and hoarseness (Table 1). The symptoms began a few hours to one day postoperatively. The postoperative physical examination revealed atrophy and deviation of the tongue in most cases [13,14,18,19,21,22,23,24].

Video-fluoroscopic swallowing was performed in five cases to rule out pathological causes in the pharynx and esophagus, such as obstruction [13,18,19,21,22]. Lee et al. [19], Colón et al. [18], and Park et al. [22] found an impaired oral phase, delayed bolus formation, reduced tongue motility, and pooling of saliva using video-fluoroscopic swallowing. Moreover, they emphasized the importance of diagnosing HNP, as it may lead to other complications, such as aspiration pneumonia [18,22]. MRI of the head and neck was performed to rule out intracranial causes [18,22]. Lee et al. [19] found atrophy and signal changes of the tongue on MRI. Nerve conduction studies were performed in two cases to confirm hypoglossal neuropathy and denervation [18,22]. Colón et al. [18] confirmed left hypoglossal neuropathy, and Park et al. [22] found a positive sharp wave and reduced recruitment of the right tongue and cricothyroid muscles.

Five cases in the anterior approach group, including the two current cases, showed a recovery of hypoglossal nerve function at the last follow-up, which often consisted of a few months. Saunders et al. [17] did not disclose whether the patient recovered from the palsy or not. Four cases had permanent nerve injuries [13,16,18,19]. The patients experienced persistent symptoms, such as dysphagia and dysarthria. In three of the cases with permanent paralysis, the patients had undergone ACDF, and in one case ACCF (Table 1). The follow-up time varied from one month to two years.

Park et al. [22] reported complete recovery after seven months, although mild atrophy of the tongue was still present. López et al. [20] reported spontaneous and gradual improvement after ten days. Both López et al. [20] and Yasuda et al. [21] reported spontaneous recovery during rehabilitation with speech therapy and head tilt when swallowing [18,19,22].

**Table 1:** Summary of included articles about anterior cervical surgery.

Study	Study Design	Number with HNP	Indication	Procedure	Operated Levels	Injury Severity	Symptoms Post-Op	Follow-Up Time	Possible Cause	Treatment
1991 Saunders et al. [17] USA	Prospective study	1	Myelopathy	Corpectomy	NA	NA	Dysarthria Dysphagia	2 + 6 weeks, 3 + 6 months, yearly (2–5 years)	Unclear	Speech and swallowing therapy
1999 Sengupta et al. [13] UK	Case report	1	Infection	ACCF	C2–C5	Permanent	Dysarthria Dysphagia Tongue deviation	5 days 18 months	Mechanical injury	Speech therapy
2011 Park et al. [22] Republic of Korea	Case report	1	Trauma	ACDF	C3–C4	Temporary	Hoarseness Dysphagia Atrophy	7 months	Mechanical injury	NA
2015 Yasuda et al. [21] Japan	Case report	1	Dysphagia due to osteophyte	Osteophyte resection with the Smith-Robinson approach	C3–C4	Temporary	Dysarthria Dysphagia	1 year	Mechanical injury	NA
2017 Yamagata et al. [16] Japan	Retrospective study	1	Degenerative spine surgery	ACDF	NA	Permanent	Dysphagia	2/3, 7, 30 days	Unclear mechanism, possibly anatomical anomaly	NA
2017 López et al. [20] Spain	Case report	1	Trauma	Corpectomy	C3	Temporary	Dysarthria Dysphagia	1 year	Mechanical injury	NA
2020 Lee et al. [19] Republic of Korea	Case report	1	Intervertebral disc degeneration	ACDF	C3–C4	Permanent	Dysarthria Dysphagia Hemiatrophy Tongue deviation	2 years	Mechanical stress, hypotension, ischemia, neck positioning, reperfusion injuries, or surgical manipulation	NA
2020 Colón et al. [18] USA	Case report	1	Chronic neck and left arm pain	ACDF	C3–C5	Permanent	Dysarthria Dysphagia Hemiatrophy Tongue deviation	10 months 1 year Several years	Mechanical injury	NA
2023 Hellquist et al. Sweden	Current case report	1	Radiculopathy	ACDF	C5–C7	Temporary	Hoarseness Tongue deviation	2, 4, 5 months	Possibly mechanical injury from the endotracheal tube	NA
2023 Hellquist et al. Sweden	Current case report	1	Radiculopathy	ACDF	C5–C6	Temporary	Hoarseness Dysarthria Dysphagia Tongue deviation	2 months	Unclear, possibly mechanical injury from the endotracheal tube	Speech and swallowing therapy

ACDF, anterior cervical discectomy and fusion; ACCF, anterior cervical corpectomy and fusion; HNP, hypoglossus nerve palsy; NA, not available.

#### 3.2.2. HNP Following a Posterior Approach

A total of seven cases of HNP after posterior cervical spine surgery were reported. A multi-institutional retrospective study by Ames et al. [15] identified one case of HNP in 8887 performed laminectomies, posterior cervical spine procedures, and reported an incidence of 0.01%. The institutional rates may, however, vary from 0% to 1.28% [15]. The patient who had developed an HNP had undergone a laminectomy from C3 to C7 for cervical myelopathy [15].

The posterior procedures included occipitocervical fusion, atlantoaxial fusion, expansive open-door laminoplasty (ELAP), laminectomy, posterior cervical decompression and fusion (PCDF), and posterior cervical foraminotomy [14,15,23,24,25,26]. The number of operated levels varied from one to several from C0 to T2 [14,15,23,24,25,26]. The indications for surgeries were trauma [24,26] and degenerative disorders [14,15,23,25].

The most frequent possible cause of HNP after a posterior approach was anesthetic airway management. Ames et al. [15], Ishihara et al. [16], Gannon et al. [26], and Silva et al. [14] reported anesthetic airway management as a potential risk factor for mechanical injury on the nerve. Gannon et al. [26] and De Abreu et al. [23] reported no complications after endotracheal intubation. Gannon et al. [26] also mentioned the Jefferson fracture to be a possible cause. Moreover, hyperflexion of the neck was also reported as a possible risk factor [14,15]. De Abreu et al. [23] reported HNP resulting from mechanical compression caused by a misplaced C1 screw.

All patients experienced dysphagia postoperatively. Additionally, most of the patients experienced dysarthria and hoarseness. Hong et al. [24] reported pain in the tongue as an additional symptom, which originated from inadvertent tongue biting [24]. Physical examination found deviation of the tongue in most of the patients [14,23,24,25]. Ames et al. [15] did not disclose postoperative symptoms or follow-up times. Gannon et al. [26] reported a bilateral HNP.

An MRI of the head and neck was performed to rule out intracranial pathologies. No article in the posterior approach group reported any pathological findings on the MRI [23,25,26]. Laryngoscopy was performed to rule out postoperative complications related to the larynx, airways, and vocal cords [19,22,25]. A nerve conduction study was performed in a case by De Abreu et al. [23]. No electromyographic activity on the right side of the tongue was reported.

Park et al. [22], Ishihara et al. [25], and Silva et al. [14] diagnosed their patients with peripheral Tapia syndrome. The articles defined Tapia syndrome as unilateral hypoglossal and recurrent laryngeal nerve palsy. The described symptoms are dysarthria, dysphagia, and hoarseness [14,22,25], similar to our own two cases.

All of the posterior surgeries resulted in temporary HNP, and recovery was reported within a few months [14,24,26]. The follow-up times varied from two months to one year. Ishihara et al. [25] reported functional recoveries after one and two months in their two cases, both of whom were treated with vitamin B12. Other authors reported speech therapy, nutritional changes, laryngeal exercises, and nasogastric tube feeding as interventions [14,23,24,26].

**Table 2:** Summary of included articles about posterior cervical surgery.

Study	Study Design	Number with HNP	Indication	Procedure	Operated Levels	Injury Severity	Symptoms Post-Op	Follow-Up Time	Possible Cause
2022 De Abreu et al. [23] USA	Case report	1	Myelopathy	PCDF	C1–T2	Temporary	Dysphagia Tongue deviation	3 months	Mechanical injury (compression—C1 LMS)
2023 Ishihara et al. [25] Japan	Case report	2	Spinal canal stenosis	ELAP	C4–C6	Temporary	Dysarthria Dysphagia Hoarseness Tongue deviation	1 month, 2 months	Mechanical injury or anesthetic airway management
2020 Gannon et al. [26] USA	Case report	1	Trauma	Occipitocervical fusion	C0–C3	Temporary	Dysarthria Dysphagia	6 months	Anesthetic airway management or Jefferson fracture
2017 Ames et al. [15] USA	Retrospective study	1	Myelopathy	Laminectomy (posterior fusion)	C3–C7	Temporary	NA	NA	Anesthetic airway management or hyperflexion
2019 Silva et al. [14] UK	Case report	1	Bilateral upper limb radiculopathy	Posterior cervical foraminotomy	C5–C7	Temporary	Hoarseness Dysphagia Tongue deviation	1 year	Anesthetic airway management or hyperflexion
2006 Hong et al. [24] USA	Case report	1	Trauma (fracture)	Posterior atlantoaxial fixation	C1–C2	Temporary	Dysarthria Dysphagia Tongue deviation	2, 4, 8 months	Mechanical injury

LMS, lateral mass screw; PCDF, posterior cervical decompression and fusion; ELAP, expansive open-door laminoplasty; HNP, hypoglossus nerve palsy; NA, not available.

## 4. Discussion

Historically, the issue of HNP has been described in association with the submandibular retropharyngeal approach to the craniocervical junction [27]. However, our systematic review of the literature identified a total of 15 reported cases of HNP following conventional anterior or posterior cervical spine surgery. The risk of this complication was exceedingly low, varying from 0.6% to 2.5% after anterior surgery and is reported to range from 0.01% to 1.28% after posterior surgery [15,16,17].

Most of the patients were male, and the mean age was lower in the anterior approach group. A retrospective study by Harel et al. [28] reported a lower mean age of the patients in the anterior approach group compared to the posterior approach group, although no difference in risk factors was found in that study. In a prospective study by Lange et al. [29], higher age was not correlated with more complications intra- or postoperatively.

Both our patients underwent right-sided ACDF at C5–C7 or C5–C6 for right-sided radiculopathy. In contrast, previously reported anterior cases had been operated on at higher cervical levels (C3–C5) [13,18], which may suggest that HNP is more common after more cephalad surgery using the anterior approach. On the other hand, posterior surgeries were performed equally across all cervical levels.

Most of the studies, including our cases (10/17 patients), reported degenerative disorders as the indication for surgery. Trauma was reported in four cases and infection in one case. Given the limited nature of the data, it is unclear whether the incidence of HNP as a surgical complication risk may be overrepresented across a specific surgical indication for either anterior or posterior approach.

### 4.1. Etiology

The most common reason for HNP after cervical spine surgery was mechanical injury. The mechanical injury may result from excessive retraction, compression by screws or surgical staples, mechanical stress, or neck positioning. Furthermore, mechanical injury may arise from extensive dissection of neurovascular structures [2].

Another common reason for HNP is anesthetic airway management. In fact, iatrogenic hypoglossal nerve palsy is considered to be a rare but serious complication of endotracheal intubation, with reported incidence ranging between 0.36% and 2.7% [30,31,32]. This is especially relevant in posterior cervical spine surgery, where it is important to emphasize the risks of neck flexion combined with endotracheal intubation. Kang et al. [33] reported a case of Tapia’s syndrome after posterior cervical spine surgery, where airway management was thought to be the cause. The endotracheal intubation was performed without any complications, and the pressure was regularly checked. Even though the exact mechanisms behind the HNP were not fully understood, Kang et al. [33] speculated it may result from hyperflexion of the neck, which leads to mechanical stretch on the cranial nerves by the endotracheal tube. Park et al. [34] hypothesize that hyperflexion of the neck causes the endotracheal tube to bend more in the pharynx, leading to compression against the wall of the pharynx. Moreover, the bent tube may shift from the middle of the pharynx to the lateral wall where the hypoglossal and laryngeal nerves are situated close to each other [34]. Nevertheless, none of the cases reported difficulties with airway management. While the posterior approach carries a higher risk of endotracheal tube-related injury or even direct injury to the nerve due to misplaced screws, anterior surgery is notably associated with a greater risk of neural injury due to retraction of the esophagus, airways, and surrounding neurovascular structures. This is reflected in the seemingly higher reported incidence of HNP following anterior procedures compared to posterior ones. In summary, it may be said that HNP in the anterior approach is more likely due to direct mechanical compression, whereas in the posterior approach, it is more likely the result of indirect compression, such as pressure exerted by the endotracheal tube. MRI scans of the head and neck did not show any pathology in most cases, and similarly, the MRI was normal in our case. A single patient had atrophy and signal changes of the tongue on MRI [19]. EMG is also an alternative to investigate nerve function. De Abreu [23] reported low EMG activity, indicative of hypoglossal dysfunction. Laryngeal endoscopy was also performed in most of the cases to examine the airways after endotracheal intubation. In no case was pathology noted. However, hoarseness is a hallmark symptom of recurrent laryngeal nerve palsy, which may occur due to mechanical injury to the nerve during the Smith-Robinson dissection [35]. As recurrent laryngeal nerve palsy results in vocal cord paralysis, laryngeal endoscopy is important to rule out this condition. Deviation and atrophy of the tongue were observed in some cases during the postoperative examination, highlighting the importance of physical examination when the patient presents with dysarthria and dysphasia after spine surgery. A fluoroscopic swallowing examination was performed in most cases to evaluate dysphagia. It is important to evaluate and diagnose HNP to minimize the risk of complications from dysphagia, such as aspiration pneumonia [18,22].

### 4.2. Prognosis

Dysphagia is a common symptom after anterior spinal surgery [36]. A study by Bazaz et al. [36] found female sex and surgery on multiple levels to be risk factors for postoperative dysphagia. Six months postoperatively, most cases of dysphagia have resolved [36]. Tasiou et al. [35] reported no significant risk factors for dysphagia, such as sex or type of surgical procedure. A study by Tatter et al. [37] reported that approximately half of the patients who presented with dysphagia after posterior cervical spine surgery improved or had a complete recovery of the symptoms.

Most of the time, the complications and symptoms are transient and improve within a few days to months postoperatively [2,38]. Ishihara et al. [25] treated the two patients with HNP using vitamin B12 supplements. According to Okada et al. [39], vitamin B12 accelerates nerve regeneration after peripheral nerve injury. In other cases, the patients were treated with speech and swallowing therapy [14,18,19,23,26]. Two articles by Gannon et al. [26] and Hong et al. [24] reported nutritional therapy, such as nasogastric tubes, to minimize the risk of aspiration pneumonia. We suggest that all patients undergo follow-up assessments for swallowing to minimize the risk of aspiration.

Permanent HNP is more likely to occur after anterior cervical spine surgery compared to posterior. Permanent HNP was reportedly the result of mechanical injury, possibly in relation to anatomical variations and mechanical stress. Permanent HNP results in an increased risk of aspiration pneumonia. Therefore, it is important to diagnose the palsy as soon as possible to avoid this complication.

Nimodipine is a dihydropyridine calcium channel blocker that has been used in different clinical settings since 1980 [40]. The drug has several different clinical indications, such as ischemic stroke, aneurysmal subarachnoid hemorrhage, migraines, and traumatic brain injury [40]. Furthermore, nimodipine was suggested to have a neuroprotective role [40]. A systematic review and meta-analysis by Lin et al. [41] reported a significantly increased recovery of recurrent laryngeal nerve and facial nerve injuries with a monotherapy of nimodipine compared with controls. However, there are no published studies on nimodipine’s effect on HNP in humans. Further studies are needed to clarify a possible role for nimodipine in the treatment of HNP.

### 4.3. Possible Preventive Measures

Preventive measures aiming to reduce the risk of HNP primarily focus on minimizing intubation-related trauma to surrounding soft tissues, as direct prevention of surgical injury to the nerve is often influenced by less controllable factors, such as surgical technique, surgeon skill, and individual patient anatomy. In this context, several preventive strategies have been suggested based on the possible mechanisms of intubation-related nerve injury. One key approach is to use less invasive airway management techniques, such as a laryngeal mask airway (LMA) instead of an endotracheal tube (ETT), where feasible [42]. Another potential strategy is the use of video laryngoscopy during intubation to facilitate a more precise and atraumatic placement.

Additionally, some studies suggest that monitoring cuff pressure during intubation can lower the incidence of postoperative HNP [43,44]. For instance, limiting LMA cuff pressures to under 60 cm H_2_O has been shown to reduce dysphagia and dysphonia shortly after surgery [45], while maintaining ETT cuff pressures below 20 cm H_2_O has been associated with a reduced risk of sore throat following cervical spine procedures [46]. Other preventive measures include the periodic lowering of cuff pressure in prolonged surgeries, the use of pressure-relief valves, and deflating airway cuffs during patient repositioning to minimize compression risks to the hypoglossal and recurrent laryngeal nerves [47,48,49]. Surgeons and anesthesiologists should remain cautious during endotracheal intubation, ensuring proper tube placement and minimal pressure on surrounding tissues. Vigilance in managing cuff pressures and using less invasive airway techniques, when possible, can help mitigate the risk of intubation-related nerve injury. Maintaining awareness of these factors is crucial for minimizing complications, such as HNP. Additionally, intraoperative nerve monitoring may be an option for the continuous assessment of the hypoglossal nerve, particularly in complex surgical cases where a high risk of HNP may be expected [50]. Other non-specific preventive practices to mitigate HNP risk include avoiding excessive neck extension or flexion and multiple intubation attempts, intermittent patient position checks, and early consultation for patients presenting with neurological symptoms [42].

### 4.4. Strengths and Limitations

We present two case reports that, in and of themselves, provide only limited data. The study also includes a review of the literature. Unfortunately, the available literature is scarce and primarily made up of single-case reports. This results in low levels of evidence and high risks of bias. Additionally, the geographic representation of the included studies pertains to the USA, Europe, and East Asia. Therefore, the study lacks generalizability from a global perspective.

## 5. Conclusions

HNP is a rare complication following cervical spine surgery. Common features of HNP include dysarthria, dysphagia, and hoarseness. The causes of hypoglossal palsy is multifactorial, but mechanical injury is the most common cause. HNP is often transient and recovers with conservative treatment. However, it is important to recognize HNP to prevent aspiration due to poor swallowing. Further studies to elucidate the mechanisms contributing to HNP in cervical spine surgery would enable the development of preventive measures. In the meantime, prioritizing non-traumatic airway management techniques in patients undergoing cervical spine surgery may be the most effective approach to reducing this complication.

## Data Availability

The original contributions presented in this study are included in the article/Supplementary Materials. Further inquiries can be directed to the corresponding author.

## Supplementary Materials

The following supporting information can be downloaded at: https://www.mdpi.com/article/10.3390/brainsci15030256/s1, Table S1: Conducted search strategy.

## Abbreviations

The following abbreviations are used in this manuscript:

ACCF	Anterior Cervical Corpectomy and Fusion
ACDF	Anterior Cervical Discectomy and Fusion
ELAP	Expansive Open-Door Laminoplasty
EMG	Electromyography
ENT	Ear, Nose, and Throat
HNP	Hypoglossal Nerve Palsy
MRI	Magnetic Resonance Imaging
PCDF	Posterior Cervical Decompression and Fusion
