# Supplementary material for: Hypoglossal Nerve Palsy Following Cervical Spine Surgery—Two Case Reports and a Systematic Review of the Literature
Source: Brain Sci. 2025 Feb 27;15(3):256. doi: 10.3390/brainsci15030256 (PMC11940092; doi:10.3390/brainsci15030256)
Supplement: Supplementary file 1 [file brainsci-15-00256-s001.zip › brainsci-3494681-supplementary.pdf]

Table S1. Conducted search strategy

Search	Query	Results
#1	"Hypoglossal Nerve Injuries"[Mesh]	277
#2	(((("hypoglossal nerve injur*"[Title/Abstract]) OR ("hypoglossal nerve pals*"[Title/Abstract])) OR ("hypoglossal nerve paresis"[Title/Abstract])) OR ("Twelfth Cranial Nerve Injur*"[Title/Abstract])) OR ("Twelfth-Nerve Pals*"[Title/Abstract])	582
#3	#1 OR #2	798
#4	"Surgical Procedures, Operative"[Mesh]	3.574.448
#5	"Postoperative Complications"[Mesh:NoExp]	403.530
#6	surgery[Title/Abstract] OR fixation[Title/Abstract] OR fusion[Title/Abstract] OR decompression[Title/Abstract] OR lamin*[Title/Abstract] OR corpectom*[Title/Abstract] OR discectom*[Title/Abstract]	2.009.726
#7	#4 OR #5 OR #6	4.729.687
#8	#3 AND #7	304
